# Identification of Genetic Networks Reveals Complex Associations and Risk Trajectory Linking Mild Cognitive Impairment to Alzheimer’s Disease

**DOI:** 10.3389/fnagi.2022.821789

**Published:** 2022-02-17

**Authors:** Claudia Strafella, Valerio Caputo, Andrea Termine, Carlo Fabrizio, Giulia Calvino, Domenica Megalizzi, Paola Ruffo, Elisa Toppi, Nerisa Banaj, Andrea Bassi, Paola Bossù, Carlo Caltagirone, Gianfranco Spalletta, Emiliano Giardina, Raffaella Cascella

**Affiliations:** ^1^Genomic Medicine Laboratory, IRCCS Santa Lucia Foundation, Rome, Italy; ^2^Medical Genetics Laboratory, Department of Biomedicine and Prevention, Tor Vergata University, Rome, Italy; ^3^Laboratory of Experimental Neuropsychobiology, Department of Clinical and Behavioral Neurology, IRCCS Santa Lucia Foundation, Rome, Italy; ^4^Laboratory of Neuropsychiatry, Department of Clinical and Behavioral Neurology, IRCCS Santa Lucia Foundation, Rome, Italy; ^5^Department of Clinical and Behavioral Neurology, IRCCS Fondazione Santa Lucia, Rome, Italy; ^6^Department of Biomedical Sciences, Catholic University Our Lady of Good Counsel, Tirana, Albania

**Keywords:** mild cognitive impairment, Alzheimer’s disease, genetic risk variants, biomarkers, therapeutic target

## Abstract

Amnestic mild cognitive impairment (aMCI) and sporadic Alzheimer’s disease (AD) are multifactorial conditions resulting from a complex crosstalk among multiple molecular and biological processes. The present study investigates the association of variants localized in genes and miRNAs with aMCI and AD, which may represent susceptibility, prognostic biomarkers or multi-target treatment options for such conditions. We included 371 patients (217 aMCI and 154 AD) and 503 healthy controls, which were genotyped for a panel of 120 single nucleotide polymorphisms (SNPs) and, subsequently, analyzed by statistical, bioinformatics and machine-learning approaches. As a result, 21 SNPs were associated with aMCI and 13 SNPs with sporadic AD. Interestingly, a set of variants shared between aMCI and AD displayed slightly higher Odd Ratios in AD with respect to aMCI, highlighting a specific risk trajectory linking aMCI to AD. Some of the associated genes and miRNAs were shown to interact within the signaling pathways of APP (Amyloid Precursor Protein), ACE2 (Angiotensin Converting Enzyme 2), miR-155 and PPARG (Peroxisome Proliferator Activated Receptor Gamma), which are known to contribute to neuroinflammation and neurodegeneration. Overall, results of this study increase insights concerning the genetic factors contributing to the neuroinflammatory and neurodegenerative mechanisms underlying aMCI and sporadic AD. They have to be exploited to develop personalized approaches based on the individual genetic make-up and multi-target treatments.

## Introduction

In the last decades, several research efforts have been made to dissect the complex scenario underlying neurodegeneration, with a particular attention to mild cognitive impairment (MCI) and Alzheimer’s disease (AD). In particular, amnestic MCI (aMCI) is the prodromal stage of AD and is characterized by memory deficits, often associated with deterioration of other cognitive abilities (Albert et al., 2011), and with quite intact activities of daily living. Patients with aMCI have a high risk to rapidly progress toward AD, whereas patients suffering from non-amnestic MCI (naMCI) may progress to other forms of dementias such as Frontotemporal Dementia or Dementia with Lewy Bodies (Bondi et al., 2017; Tao et al., 2020). Structural/pathological brain features of AD are neuron loss in the hippocampus and neocortex/entorhinal cortex, and atrophy of temporal and parietal cortex (Yacoubian, 2017). Additional neuropathological hallmarks of AD are the presence of extracellular β-amyloid (Aβ) plaques and intracellular neurofibrillary tangles (NFTs) resulting from the deregulation of Amyloid Precursor Protein (APP) and the increasing of Phosphorylated-Tau (P-Tau) protein, respectively (Yacoubian, 2017). MCI and AD are preceded by a long asymptomatic, preclinical, phase that may start as long as 20 years before the appearance of the first symptoms, with the occurrence of brain changes, synaptic dysfunction, synapse loss and amyloid buildup (Yacoubian, 2017; Bottero and Potashkin, 2019; DeTure and Dickson, 2019). For many years, the so-called “amyloid hypothesis” provided a view of AD as a neuron-centric, linear model of disease initiated by Aβ deposition and followed by a cascade of abnormal events that, ultimately, led to progressive neurodegeneration (De Strooper and Karran, 2016; Selkoe and Hardy, 2016). Although this model represented an important milestone for investigating potential diagnostic hallmarks and drug targets for AD, the extensive progress in the field of biomedicine put in question the linearity of the “amyloid hypothesis” as the primary causative mechanism of disease (De Strooper and Karran, 2016; Selkoe and Hardy, 2016). It is nearly apparent that the neuron-centric “amyloid hypothesis” is probably just a part of a more compound story, which includes different cell types interacting together, several genetic determinants and diverse biological pathways affecting both aging and neurodegenerative conditions (De Strooper and Karran, 2016; Tábuas-Pereira et al., 2020). Although the allele *epsilon 4* of *APOE* (*Apolipoprotein E*) gene represents the major risk variant for sporadic AD, the Genome-Wide Association Studies (GWAS) have identified several genetic variants conferring a small but significant risk to develop AD and MCI. These variants have been localized in the proximity of several genes, including *SORL1* (*Sortilin Related Receptor 1*), *BIN1* (*Bridging Integrator 1*), *CR1* (*Complement C3b/C4b Receptor 1-Knops Blood Group*), *CLU* (*Clusterin*), *PICALM* (*Phosphatidylinositol Binding Clathrin Assembly Protein*), *ABCA7* (*ATP Binding Cassette Subfamily A Member 7*), *MS4A* (*Membrane Spanning 4-Domains A10*)-cluster, *TOMM40* (*Translocase Of Outer Mitochondrial Membrane 40*), *TREM2* (*Triggering Receptor Expressed On Myeloid Cells 2*), *ADAM10* (*ADAM Metallopeptidase Domain 10*) and many others (Verheijen and Sleegers, 2018; Fan et al., 2019; Sierksma et al., 2020; Tábuas-Pereira et al., 2020). To date, several biological pathways (transport and metabolism of lipids, intracellular vesicular trafficking, immuno-inflammatory response, apoptosis, synaptic failure, oxidative stress, calcium metabolism, iron homeostasis, mitochondrial dysfunction) have been proposed as driver mechanisms of AD and MCI (Verheijen and Sleegers, 2018). However, the extent to which the genetic background or genomic architecture, the biological pathways other than Aβ cascade or Tau pathology eventually shapes the risk and the trajectory of disease in individuals are still under debate. Given these premises, the present study aimed at investigating the association of variants localized within genes and miRNAs that could enhance the knowledge of the genetic factors contributing to the susceptibility and pathophysiology of aMCI and AD.

## Materials and Methods

### Selection of Genetic Variants

The study was performed utilizing a panel of 120 variants available from two previous studies (Strafella et al., 2021a,b) conducted on other complex disorders, which are known to share some disease mechanisms with MCI and AD. The genetic variants of interest have been selected in relation to their location within or nearby genes primarily involved in cellular homeostasis, inflammation, immune response, signal transduction, neuronal development and functioning, synaptogenesis as well as genes known to be involved in AD, MCI and other complex diseases characterized by neurodegeneration (namely, Parkinson’s Disease and Multiple Sclerosis). A detailed description of the methods utilized for selecting the variants of interest is available within the referenced paper and its Supplementary Material (Strafella et al., 2021a).

The existence of Linkage Disequilibrium (LD) patterns among variants located on the same chromosomes was evaluated in European samples derived from 1,000 Genomes database. LD analysis was performed through the LDmatrix tool of LDlink software (Machiela and Chanock, 2015), obtaining a heatmap matrix representing the LD patterns among the variants for each chromosome. Moreover, D’ and R2 values have been obtained for each pairwise LD, as well. Considering the variants located in chromosome 1, high LD scores were obtained for rs2300747-rs1335532; rs1772159-rs823137; rs786843-rs1505067. On chromosome 7, LD was detected for rs2280714-rs10954213. On chromosome 8, high LD was reported for rs9331896-rs11136000. Concerning chromosome 10, rs12722489-rs2075650 revealed total LD. On chromosome 20, rs2248359-rs2248137 revealed high LD. Finally, the rs1137070-rs2072743 variants located on chromosome X reported high LD value.

### Study Subjects

The study cohort involved 371 Italian unrelated patients recruited from the Outpatient Memory Clinic of the Laboratory of Neuropsychiatry of IRCCS Santa Lucia Foundation in Rome (Italy) in the time range included from 2010 to 2021. The patients’ cohort consisted of 154 patients with sporadic AD and 217 patients with aMCI. The sample size of both cohorts were also calculated by the one-sample proportion test in order to check if they were enough to find even the less common variants and reduce the possible biases given by low sample size. To this purpose two one-sample proportions test with continuity correction were computed to obtain a 95% Confidence Interval (CI) estimate of population proportion and calculate the sample size. Every CI was calculated using the point estimate of the Minor Allele Frequency (MAF) of the SNP showing the lowest frequency distribution. The expected frequency of the European population was retrieved using Ensembl database. At 95% confidence level, the true MAF frequency of the less common variant (rs11218343) was expected to lie between 0.03 and 0.08, with a Margin of Error (ME) of 0.03 for AD and between 0.03 and 0.07, with a Margin of Error (ME) of 0.02 for MCI. Given these results, the minimum sample size required for the present study was 138 subjects for AD and 130 for MCI (considering a 95%CI and the calculated ME), showing thereby that the size of both patient’s cohorts were appropriate for further processing.

Diagnosis of aMCI was made according to established criteria by trained neurologists who interviewed patients and next-ok-kin (Petersen and Negash, 2008; Petersen et al., 2014). Inclusion criteria for subjects in the aMCI group were the following: (1) subjective memory impairment corroborated by an assistant and confirmed by a score below the normality cut-off on one episodic memory test of the neuropsychological screening battery; (2) lack of fulfillment of NIH-NIA (National Institutes of Health-National Institute on Aging) criteria for AD (McKhann et al., 2011); (3) absence or very mild impact of the memory deficit on the activities of daily living, as confirmed by a normal score on IADL (Instrumental activities daily living) and by a total CDR (Clinical Dementia Rating) score = 0.5, consistent with a minimal change in the patient’s habits; (4) lack of any evidence indicative of neurological or systemic disorders able to induce memory deficits, as confirmed by non-pathological findings for thyroid functioning, vitamin B12, folic acid levels and internal medicine and neurological examination. MR (Magnetic Resonance) brain imaging was also negative for focal lesions (minimal diffuse changes or minimal lacunar lesions of white matter were allowed) as computed according to the semi-automated method recently published by our group (Spalletta et al., 2020). These criteria were used by the staff physicians to produce a diagnosis of MCI. To identify a homogenous group of MCI patients, and reduce the possibility of including a heterogeneous syndrome with non-AD related etiologies, MCI patients with Major Depressive Disorder were excluded if meaningful clinical improvement in cognition (defined as no longer fulfilling MCI criteria) accompanying improvement in depression was observed within 6 months of antidepressant treatment initiation. Finally, a thorough clinical examination was used to exclude patients with cognitive deficits secondary to underlying somatic disorders such as unbalanced diabetes, heart disease, or other major medical illnesses that could cause cognitive impairment.

Patients with AD met the clinical criteria for Alzheimer’s dementia established by the National Institute on Aging and the Alzheimer’s Association (McKhann et al., 2011). Their medical history, neurological examination, brain imaging and laboratory tests confirmed that the dementia symptoms were indicative of sporadic AD.

The detailed patients’ characteristics are summarized in Table 1. In patients with positive family history for AD, the presence of known pathogenic mutations associated with monogenic forms of AD was excluded. The research was approved by the Ethical committee (CE/PROG.650 approved on 01/03/2018) of IRCCS Santa Lucia Foundation Hospital of Rome and was performed according to the Declaration of Helsinki. Written informed consent was obtained for all patients. As reference group, 503 samples representative of the European general population were retrieved from 1,000 Genomes databases.

**TABLE 1:** Characteristics of patients with aMCI and sporadic AD.

Subjects	Gender (F:M)	Age	Scholarity (years)	Age of Onset	MMSE	IADL	ADL	NPI	Familiarity: neurological diseases (%)	Familiarity: psychiatric diseases (%)
aMCI (*N* = 217)	52:48	70.74 ± 7.74	10.29 ± 4.36	68.86 ± 9.71	27.45 ± 1.96	7.59 ± 2.69	6.29 ± 0.82	11.20 ± 8.86	37.8	10.4
AD (*N* = 154)	66:34	74.9 ± 7.55	8.44 ± 4.38	71.78 ± 7.65	21.20 ± 3.94	13.95 ± 5.73	8.50 ± 2.95	21.99 ± 13.36	34.9	16.7

*Detailed information about the patients recruited for the study are summarized.*

*Mean ± Standard Deviation (SD) are shown for all the features except for familiarity, which has been reported as percentage in the cohort.*

*The overall cognitive functions were measured by Mini-Mental State Examination (MMSE).*

*Patient’s functional abilities in daily living were measured by Activities of Daily Living (ADL) and Instrumental Activities of Daily Living (IADL).*

*Psychopathology-related and behavioral symptoms were measured by means of Neuropsychiatric Inventory (NPI) scale.*

*aMCI, amnestic Mild Cognitive Impairment; AD, Alzheimer’s disease.*

### DNA Extraction and Quantification

Genomic DNA was extracted from 200–400 μL of whole blood with MagPurix Blood DNA Extraction Kit and MagPurix Automatic Extraction System (Resnova, Italy) according to the manufacturer’s instructions. The concentration and quality of the extracted DNA have been assessed by DeNovix Spectrophotometer (Resnova, Italy). In particular, DNA samples reported a concentration range of 50–150 ng/μL and A260/230 and A260/280 ratios included between 1.7 and 1.9.

### Genotyping Analysis

Firstly, the *APOE* genotype was assessed in the patient’s cohort. To this purpose, Real-Time PCR and Taqman Genotyping assay was utilized to screen the patients for the rs7412 (C/T) and rs429358 (T/C) polymorphisms, the haplotype of which determine the *APOE* genotype. In particular, the possible *APOE* genotypes (ε*1*, ε*2*, ε*3* or ε*4*) were classified according to the following allele combinations (ε*1*: rs7412_T/rs429358_C; ε*2*: rs7412_T/rs429358_T; ε*3*: rs7412_C/rs429358_T; ε*4*: rs7412_C/rs429358_C).

Successively, the DNA samples were subjected to a massive genotyping performed by OpenArray Real-Time PCR technology on Quant Studio 12K Flex Real Time PCR System (Thermo Fisher Scientific, CA, United States). Open Array technology employs TaqMan OpenArray plates with 3,072 through-holes, in which the Taqman probes (Thermo Fisher Scientific, CA, United States) are spotted. The customized panel of 120 assays designed for the selected variants enabled the simultaneous genotyping of 24 DNA samples per plate. For each sample, 30–150 ng of extracted DNA have been re-suspended in 3 μL of pure distilled water and manually loaded into 384 well-plates together with 3 μL of TaqMan OpenArray Genotyping Master Mix according to manufacturer’s instructions. Negative controls were obtained by combining water and Master Mix in a 1:1 ratio. The obtained mix have been automatically transferred on the TaqMan OpenArray plates through the QuantStudio 12K Flex Accufill System. The loaded plates have then been inserted into the QuantStudio 12K Flex Real Time PCR system (Thermo Fisher Scientific, CA, United States) to perform the Real-Time PCR run. Results have been analyzed by the Taqman Genotyper Software (Thermo Fisher Scientific, CA, United States) that enabled to perform the genotypes calling and the quality control. In particular, cluster normalization was performed with default parameters to normalize run-to-run variations in cluster positions caused by differences in reagent lots and experimental conditions. After normalization, the call rate (defined as the percentage of successful calls) was evaluated for each SNP considering a cut-off of 90%. Therefore, the SNP assays that failed to reach this threshold were excluded from further analyses. The removed variants were rs45596840, rs6811520, rs786843, rs20417, rs17174870, rs356219 and rs2672603 for aMCI cohort; and rs786843, rs4648356, rs45596840, rs6811520, rs20417, rs356219, rs17174870, rs6964, rs2925980, rs2672603 for AD group.

### Statistical Analysis

All the statistical analyses were performed with R software (v. 4.0.3) and packages (R Core Team, 2020). Hardy-Weinberg Equilibrium (HWE) for the study cohort was tested by means of two sided Fisher’s Exact test at each locus by comparing the observed genotype frequencies with those expected under HWE. The resulting data were considered in HWE with *p-value* (*p*) > 0.05. Two association studies implementing a case-control design (AD *vs.* CTR; aMCI *vs.* CTR) were conducted to assess the differences between allele and genotype frequencies. Genotype data were analyzed using multiple Two-sided Fisher’s Exact Tests and alleles and genotypes Odds Ratio (OR), with estimation of 95% confidence intervals. The significance threshold was set at *p* < 0.05 and the obtained p were adjusted for False Discovery Rate (FDR) by calculating the *q-value* (*q*) and setting the significance threshold at *q* < 0.05 (Storey et al., 2021). In the present study, only significant data passing the *q* threshold were considered and subjected to further statistical and bioinformatic analysis. Concerning the assessment of *APOE* genotype, patients and controls were stratified in *APOE-ε4* carriers and non-carriers, in order to test the ability of this genotype to discriminate AD and aMCI cases vs. the reference group. To this purpose, r:vcd package (v.1.4–8) was utilized to test the classification model.

LD patterns among the associated variants were evaluated based on the location within the same chromosome. The LD and haplotype analyses were performed on Haploview 4.2 (Barrett et al., 2005) with default parameters, and D’ and R2 scores were obtained for each pairwise LD. The SNP showing significant differences in the allelic frequency distributions between cases (AD or MCI) and reference samples (CTR) (namely AD/CTR and aMCI/CTR) were used as input data for two Machine Learning (ML) classifiers, computing the evaluation metrics [namely, Area Under the Curve of the Receiver Operating Characteristic (AUC-ROC), accuracy, sensitivity, and specificity] both on the cross-validation and the test set.

For the AD/CTR and aMCI/CTR classifiers, AD and aMCI subjects with more than 40% of missing values in the dataset were removed while the remaining missing values were imputed using a Linear Discriminant Analysis (LDA) approach in the MICE (Multivariate Imputation by Chained Equation) package (Zhang, 2016). The data were split into train and test sets and fed to a set of 13 ML classifiers with 5 different resampling strategies pipelines. The set of models included Logistic Regression, Bayesian Generalized Linear Model (BGLM), Elastic Net, LogitBoost, Logic Regression, Support Vector Machine with linear and radial kernel, Binary Discriminant Analysis, Naive Bayes, Classification Tree, Random Forest, Bagged CART (Classification And Regression Tree), Stochastic Gradient Boosting (GBM). Resampling strategies included none, up-sampling, down-sampling from Caret package (v. 6.0–86) (Kuhn, 2015), ROSE (Random Over-Sampling Examples, v. 0.0–3) (Lunardon et al., 2014) and Smote in DMwR package (v. 0.4.1) (Chawla et al., 2002). For AD/CTR classifier, a BGLM trained on up-sampled data during k-fold cross-validation (*k* = 5, folds = 10) was selected as the final model based on the evaluation metrics computed on the independent test set. For the MCI/CTR classifier, a Random Forest model trained on down-sampled data during the repeated k-fold cross-validation (*k* = 5, folds = 10) was selected as the final model based on the evaluation metrics computed on the independent test set. In addition, variable importance measure from Random forest were also obtained, to select the most relevant predictive variables for MCI risk.

Moreover, the ORs of the SNPs associated with both aMCI and AD have been tested with a one-sample proportion test to assess if the probability of observing lower ORs in aMCI compared to AD was different from chance.

### Bioinformatic Analysis

Bioinformatic analysis investigated the possible interaction among the genes and miRNAs harboring the associated variants in the context of biological pathways relevant to the physiopathology of aMCI and AD conditions. Considering that some associated variants were located within miRNA genes, the TargetScanHuman (Agarwal et al., 2015) and miRPathDB (Kehl et al., 2020) tools were utilized to identify which of the associated genes were targeted by the miRNAs of interest and link them with specific biological pathways involved in MCI and AD. Furthermore, Ingenuity Pathway Analysis (IPA) software (Qiagen, CA, United States) was utilized to investigate interactions among genes, miRNAs and pathways characterizing the complex biological matrix underlying MCI and AD pathophysiology. IPA is an all-in-one web-based software application that allow the analysis and integration of different kinds of genetic data, facilitating their interpretation, the identification of specific targets or candidate biomarkers and placing them in the context of larger biological or chemical systems. The software is backed by the Ingenuity Knowledge Base, which consists of highly structured, detail-rich biological and chemical findings. In general, all the results generated by IPA software are referred as significant on the base of the significance enrichment score fixed at *p* < 0.05 that is calculated by Fisher’s Exact Test. In particular, Upstream Analyses, Disease and Functions and Path Designer IPA tools were employed in this study. The Upstream Analyses were utilized to identify the candidate genes taking part into signaling pathways specifically involved in biological pathways relevant to AD and/or MCI. The Disease and Functions tool was employed to categorize the genes associated with AD and MCI into specific pathophysiological pathways, functions or diseases. In this case, literature data were coupled with results retrieved from Disease and Function tool, in order to provide a more comprehensive visualization of the relationship among the associated genes, cellular and molecular pathways and diseases of interest. To this purpose, the Path Designer tool was exploited to depict the interaction among genes, miRNAs and their related targets.

## Results

### *APOE* Assessment and Association Analysis

The assessment analysis of *APOE* alleles (*ε1*, *ε2*, *ε3* or *ε4*), revealed the presence of different frequency distributions among sporadic AD cases, aMCI and control subjects. As expected, *ε1* was not found in all cohorts. In AD patients, the *APOE* alleles presented the following frequency distributions *ε2*: 1.5%, *ε3*: 75.5%, *ε4*: 23%, whereas in aMCI cases, the frequencies of *APOE* alleles (*ε2*: 6.4%, *ε3*: 80.1%, *ε4*: 13.5%) resembled those ones of controls (*ε2*: 8%, *ε3*: 78%, *ε4*: 14%). Supporting these results, the classification model performed by the r:vcd package revealed that *APOE*-*ε4* genotype was able to significantly discriminate AD cases from controls (*p* = 6.00 × 10^–4^) but could not distinguish aMCI cases from controls (*p* = 0.64).

### Statistical Association Analysis

**FIGURE 1:**
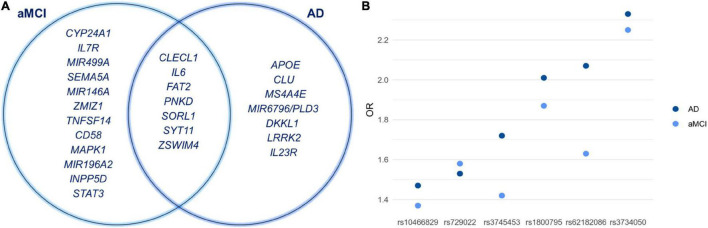
**(A)** Venn diagram showing shared and specific genes associated with aMCI and sporadic AD conditions. **(B)** The graph illustrates the ORs of the shared variants that are slightly higher in AD patients with respect to aMCI group. Although the rs11218343 is shared between aMCI and AD, it cannot be included in the graph because the OR is not available.

The statistical association analysis reported 21 SNPs and 13 SNPs associated with aMCI and sporadic AD, respectively. Tables 2, 3 report the association results of the SNPs, which passed the fixed *p* and *q* thresholds and were thereby considered for further analysis. In particular, the association analysis revealed a set of variants shared between aMCI and AD (Figure 1A), which displayed slightly higher ORs in AD cohort with respect to aMCI group (Figure 1B). This data was found to be statistically significant (*p* < 1.00 × 10^–3^) according to the one sample proportion test, suggesting that aMCI patients carrying these shared variants could be more susceptible to develop AD.

**TABLE 2:** Genetic variants significantly associated with aMCI.

SNP (*Gene*)	Variant type	Allele count in cases (Frequency)	Allele count in controls (Frequency)	*p-value*	*q-value*	OR (95%CI)
rs1800795 (*IL6*)	Intron	C: 118 (0.276) G: 310 (0.724)	C: 418 (0.416) G: 588 (0.584)	5.06 × 10^–7^	8.08 × 10^–6^	*G* = 1.87 (1.46–2.39)
rs1077667 (*TNFSF14*)	Intron	C: 372 (0.873) T: 54 (0.127)	C: 776 (0.771) T: 230 (0.229)	6.39 × 10^–6^	8.76 × 10^–5^	*C* = 2.04 (1.48–2.82)
rs9891119 (*STAT3*)	Intron	A: 311 (0.748) C: 105 (0.252)	A: 637 (0.633) C: 369 (0.367)	2.59 × 10^–5^	3.06 × 10^–4^	*A* = 1.72 (1.33–2.22)
rs2248359 (*CYP24A1*)	Regulatory region	C: 203 (0.472) T: 227 (0.528)	C: 597 (0.593) T: 409 (0.407)	2.87 × 10^–5^	3.06 × 10^–4^	*T* = 1.63 (1.29–2.06)
rs2300747 (*CD58*)	Intron	A: 380 (0.927) G: 30 (0.073)	A: 863 (0.858) G: 143 (0.142)	2.29 × 10^–4^	2.20 × 10^–3^	*A* = 2.10 (1.39–2.16)
rs12722489 (*IL2RA*)	Intron	C: 399 (0.924) T: 33 (0.076)	C: 863 (0.858) T: 143 (0.142)	4.18 × 10^–4^	3.37 × 10^–3^	*C* = 2.00 (1.35–2.98)
rs3734050 (*FAT2*)	Intron	C: 408 (0.949) T: 22 (0.051)	C: 897 (0.892) T: 109 (0.108)	4.24 × 10^–4^	3.37 × 10^–3^	*C* = 2.25 (1.40–3.62)
rs11218343 (*SORL1*)	Intron	T: 404 (1.000) C: 0 (0.000)	T: 963 (0.957) C: 43 (0.043)	4.76 × 10^–7^	8.08 × 10^–6^	*T* = na
rs729022 (*SYT11*)	3′UTR	C: 104 (0.245) T: 320 (0.755)	C: 341 (0.339) T: 665 (0.661)	4.57 × 10^–4^	3.37 × 10^–3^	*T* = 1.58 (1.22–2.04)
rs2283792 (*MAPK1*)	Intron	T: 164 (0.380) G: 268 (0.620)	T: 483 (0.480) G: 523 (0.520)	5.15 × 10^–4^	3.53 × 10^–3^	*G* = 1.51 (1.20–1.90)
rs2910164 (*MIR146A*)	Mature miRNA	C: 136 (0.315) G: 296 (0.685)	C: 231 (0.230) G: 775 (0.770)	9.51 × 10^–4^	6.08 × 10^–3^	*G* = 1.54 (1.19–1.99)
rs35349669 (*INPP5D*)	Intron	C: 272 (0.633) T: 158 (0.367)	C: 543 (0.540) T: 463 (0.460)	1.13 × 10^–3^	6.80 × 10^–3^	*C* = 1.47 (1.16–1.85)
rs2248137 (*CYP24A1*)	Intron	C: 212 (0.505) G: 208 (0.495)	C: 599 (0.595) G: 407 (0.405)	1.87 × 10^–3^	1.05 × 10^–2^	*G* = 1.44 (1.14–1.83)
rs1505067 (*SEMA5A*)	3′UTR	C: 195 (0.458) T: 231 (0.542)	C: 374 (0.372) T: 632 (0.628)	2.59 × 10^–3^	1.38 × 10^–2^	*T* = 1.42 (1.12–1.80)
rs3746444 (*MIR499A*)	Mature miRNA	A: 314 (0.737) G: 112 (0.263)	A: 811 (0.806) G: 195 (0.194)	4.77 × 10^–3^	2.26 × 10^–2^	*G* = 1.48 (1.12–1.95)
rs6897932 (*IL7R*)	Missense	C: 342 (0.799) T: 86 (0.201)	C: 733 (0.729) T: 273 (0.271)	5.10 × 10^–3^	2.26 × 10^–2^	*C* = 1.48 (1.12–1.95)
rs1250550 (*ZMIZ1*)	Intron	C: 327 (0.775) A: 95 (0.225)	C: 706 (0.702) A: 300 (0.298)	5.26 × 10^–3^	2.26 × 10^–2^	*C* = 1.46 (1.12–1.91)
rs11614913 (*MIR196A2*)	Mature miRNA	C: 278 (0.668) T: 138 (0.332)	C: 593 (0.589) T: 413 (0.411)	5.90 × 10^–3^	2.36 × 10^–2^	*C* = 1.40 (1.10–1.78)
rs10466829 (*CLECL1*)	Intron	G: 185 (0.434) A: 241 (0.566)	G: 515 (0.512) A: 491 (0.488)	7.81 × 10^–3^	2.99 × 10^–2^	*A* = 1.37 (1.08–1.73)
rs3745453 (*ZSWIM4*)	3′UTR	A: 303 (0.750) G: 101 (0.250)	A: 683 (0.679) G: 323 (0.321)	8.48 × 10^–3^	3.13 × 10^–2^	*A* = 1.42 (1.09–1.84)
rs62182086 (*PNKD*)	Intron	A: 384 (0.910) G: 38 (0.090)	A: 866 (0.861) G: 140 (0.139)	1.07 × 10^–2^	3.68 × 10^–2^	*A* = 1.63 (1.12–2.39)

*OR, odd ratio; CI, confidence interval.*

**TABLE 3:** Genetic variants significantly associated with sporadic AD.

SNP (*Gene*)	Variant type	Allele count in cases (Frequency)	Allele count in controls (Frequency)	*p-value*	*q-value*	OR (95%CI)
rs1800795 (*IL6*)	Intron	C: 80 (0.261) G: 226 (0.739)	C: 418 (0.416) G: 588 (0.584)	8.43 × 10^–7^	9.96 × 10^–6^	*G* = 2.01 (1.5–2.6)
rs3745453 (*ZSWIM4*)	3′UTR	A: 229 (0.784) G: 63 (0.216)	A: 683 (0.679) G: 323 (0.321)	4.72 × 10^–4^	4.18 × 10^–3^	*A* = 1.72 (1.26–2.34)
rs62182086 (*PNKD*)	Intron	A: 282 (0.928) G: 22 (0.072)	A: 866 (0.861) G: 140 (0.139)	1.43 × 10^–3^	1.01 × 10^–2^	*A* = 2.07 (1.30–3.31)
rs3734050 (*FAT2*)	Intron	C: 287 (0.950) T: 15 (0.050)	C: 897 (0.892) T: 109 (0.108)	1.63 × 10^–3^	1.05 × 10^–2^	*C* = 2.33 (1.33–4.05)
rs729022 (*SYT11*)	3′UTR	C: 70 (0.250) T: 210 (0.750)	C: 341 (0.339) T: 665 (0.661)	4.70 × 10^–3^	2.30 × 10^–2^	*T* = 1.53 (1.13–2.07)
rs11218343 (*SORL1*)	Intron	T: 284 (1.000) C: 0 (0.000)	T: 963 (0.957) C: 43 (0.043)	3.69 × 10^–5^	3.74 × 10^–4^	*T* = na
rs10466829 (*CLECL1*)	Intron	G: 120 (0.417) A: 168 (0.583)	G: 515 (0.512) A: 491 (0.488)	4.95 × 10^–3^	2.30 × 10^–2^	*A* = 1.47 (1.12–1.93)
rs2303759 (*DKKL1*)	Missense	T: 205 (0.679) G: 97 (0.321)	T: 765 (0.760) G: 241 (0.240)	5.50 × 10^–3^	2.30 × 10^–2^	*G* = 1.50 (1.12–2.00)
rs11136000 (*CLU*)	Intron	T: 86 (0.299) C: 202 (0.701)	T: 390 (0.388) C: 616 (0.612)	5.60 × 10^–3^	2.30 × 10^–2^	*C* = 1.49 (1.12–1.97)
rs670139 (*MS4A4E*)	Intron	G: 199 (0.696) T: 87 (0.304)	G: 611 (0.607) T: 395 (0.393)	6.84 × 10^–3^	2.55 × 10^–2^	*G* = 1.48 (1.12–1.96)
rs10889677 (*IL23R*)	3′UTR	C: 186 (0.620) A: 114 (0.380)	C: 706 (0.702) A: 300 (0.298)	8.85 × 10^–3^	2.99 × 10^–2^	*A* = 1.44 (1.09–1.90)
rs3745198 (*PLD3*)	Intron	C: 183 (0.618) G: 113 (0.382)	C: 537 (0.534) G: 469 (0.466)	1.14 × 10^–2^	3.54 × 10^–2^	*C* = 1.41 (1.09–1.84)
rs1491942 (*LRRK2*)	Intron	C: 227 (0.747) G: 77 (0.253)	C: 819 (0.814) G: 187 (0.186)	1.15 × 10^–2^	3.54 × 10^–2^	*G* = 1.49 (1.08–2.03)

*OR, odd ratio; CI, confidence interval.*

The variants associated with AD and aMCI appeared to be scattered throughout several chromosomal loci. Given this result, we evaluated the LD patterns for the SNPs located on the same chromosome in order to search for different LD patterns between cases and control samples, which may affect the susceptibility to AD and aMCI. The LD analysis did not report any significant difference between LD patterns observed in cases and control subjects, meaning that they represent independent association signals for AD and aMCI risk.

### Classification of Predictive Variables for Amnestic Mild Cognitive Impairment and Alzheimer’s Disease Susceptibility by Machine Learning Approaches

**FIGURE 2:**
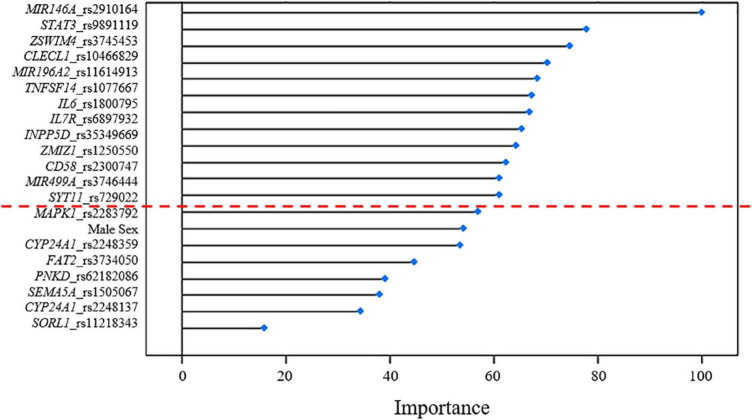
Variable importance plot showing the contribution estimated for each variant associated with aMCI to the predictive model. These estimates (reported in terms of importance score on the x-axis of the plot) are standardized to achieve a maximum score of 100. The red dashed line indicates the most relevant predictors with a score > 60.

Several machine-learning approaches have been tested in order to find the most suitable one able to identify relevant predictors of aMCI and AD risk, considering the associated SNPs, *APOE* genotype and patient’s characteristics as candidate variables. For the aMCI cases, random forest resulted to be the most appropriate over 65 tested models. The variable importance measures obtained from random forest highlighted rs2910164 (*MIR146A*, *MicroRNA 146a*), rs9891119 (*STAT3*, *Signal Transducer And Activator Of Transcription 3*), rs3745453 (*ZSWIM4*, *Zinc Finger SWIM-Type Containing 4*), rs1800795 (*IL6*, *Interleukin 6*), rs11614913 (*MIR196A2, MicroRNA 196a2*), rs1077667 (*TNFSF14*, *TNF Superfamily Member 14*), rs10466829 (*CLECL1*, *C-Type Lectin Like 1*), rs35349669 (*INPP5D*, *Inositol Polyphosphate-5-Phosphatase D*), rs2300747 (*CD58*, *CD58 Molecule*), rs6897932 (*IL7R*, *Interleukin 7 Receptor*), rs1250550 (*ZMIZ1*, *Zinc Finger MIZ-Type Containing 1*), rs3746444 (*MIR499A*, *MicroRNA 499a*) and male sex, as the most relevant predictors for aMCI risk, showing a variable importance score > 60 (Figure 2). The assessment of quality parameters revealed that this model showed an AUC = 0.71, with a 0.74 of sensitivity and 0.68 of specificity. Concerning AD cases, Bayesian Generalized Linear Model showed to be the most performant model to assess the most predictive variables for AD risk over 52 tested models. In particular, rs1800795 (*IL6*), rs62182086 (*PNKD*, *PNKD Metallo-Beta-Lactamase Domain Containing*), rs11218343 (*SORL1*), rs3745453 (*ZSWIM4*), rs1491942 (*LRRK2*, *Leucine Rich Repeat Kinase 2*) and female sex appeared as the most significant variables for predicting AD risk (Table 4). As quality parameters, an AUC = 0.74, a sensitivity = 0.80 and a specificity = 0.69 were reported for this model.

**TABLE 4:** Regression analysis model for evaluating the predictiveness of the variants associated with sporadic AD.

Variables	*p-value*	Standard error
rs1800795 (*IL6*)	1.08 × 10^–8^	0.16
rs62182086 (*PNKD*)	4.26 × 10^–7^	0.21
rs11218343 (*SORL1*)	9.79 × 10^–6^	0.43
Female Gender	1.88 × 10^–6^	0.17
rs3745453 (*ZSWIM4*)	1.29 × 10^–5^	0.16
rs1491942 (*LRRK2*)	0.0007	0.17
rs10889677 (*IL23R*)	0.0004	0.16
rs3745198 (*PLD3*)	0.0003	0.17
rs729022 (*SYT11*)	0.008	0.16
rs2303759 (*DKKL1*)	0.008	0.16
*APOE-ε4*	0.005	0.17
rs670139 (*MS4A4E*)	0.004	0.16
rs3734050 (*FAT2*)	0.004	0.23
rs11136000 (*CLU*)	0.07	0.16
rs10466829 (*CLECL1*)	0.13	0.20

### Bioinformatic Analysis

**FIGURE 3:**
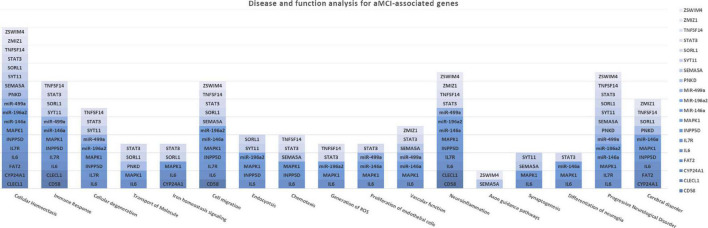
Disease and Function Analysis for the genes associated with aMCI. The figure illustrates the cellular and molecular functions mainly associated with the genes of interest.

**FIGURE 4:**
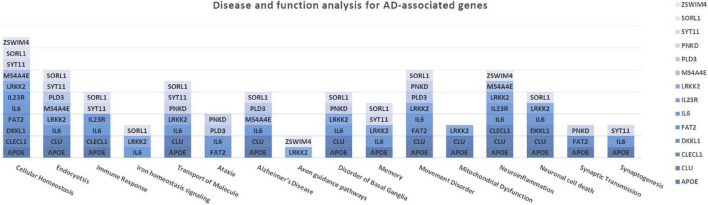
Disease and Function Analysis for the genes associated with AD. The figure illustrates the cellular and molecular functions mainly associated with the genes of interest.

**FIGURE 5:**
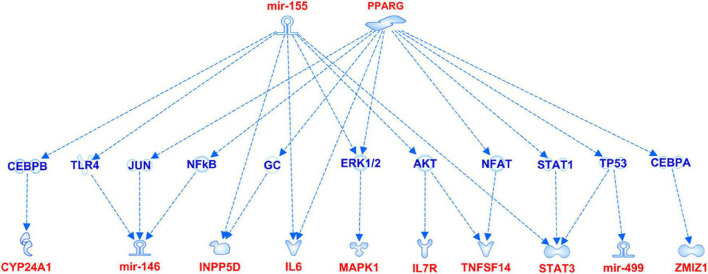
miR-155 and PPARG signaling pathways showing the interaction with the genes associated with aMCI.

**FIGURE 6:**
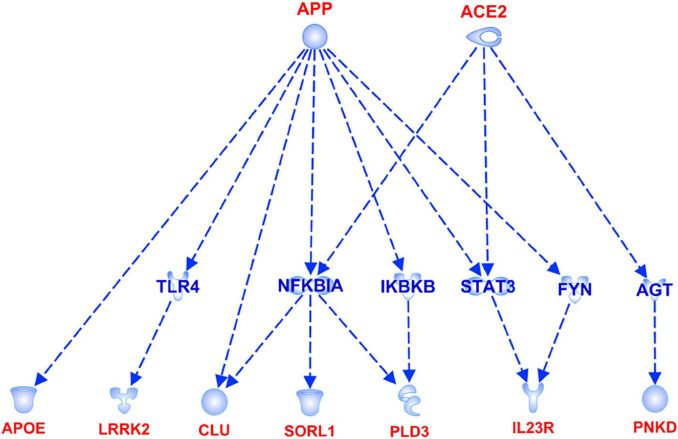
APP and ACE2 signaling pathways showing the interaction with the genes associated with AD.

The association analysis put in evidence common and shared genes associated with aMCI and AD (Figure 1), consistent with the existence of a link between these two conditions. The “Disease and Function” analysis allowed gathering the genes into specific pathways and visualize them in the context of pleiotropic cellular and molecular functions on the one hand, and of neurological functions and disorders, on the other hand. As a result, the aMCI-associated genes were primarily implicated into the regulation of pleiotropic biological pathways that affect the maintenance of cellular homeostasis and the response to aging and exogenous stress, although they were also linked to neuroinflammation, neuron wiring mechanisms, cerebral disorders and progressive neurological disorders (Figure 3). As for AD-associated genes, they were associated with cellular/molecular functions and disease conditions that are more specific to the neurological area (Figure 4). In fact, specific association with diseases mediated by neurodegeneration (i.e., Alzheimer’s disease, Movement disorders, Ataxia, disorders of basal ganglia) and functions involved in neuroinflammation, neuronal death, miswiring and mitochondrial dysfunction have been reported. Genes involved in cellular homeostasis have also been reported in AD as well, although at a lesser extent compared to aMCI. These results are suggestive of a differential role of the associated genes in determining the susceptibility to aMCI and AD conditions. Moreover, bioinformatics analysis allowed identifying a set of genes associated with MCI as downstream regulators of miR-155 (*p* = 1.76 × 10^–5^) and PPARG (*p* = 1.36 × 10^–4^) signaling pathways (Figure 5). Concerning AD-associated genes, some of them were shown to fall within APP (*p* = 9.04 × 10^–4^) and ACE2 (*p* = 2.00 × 10^–4^) signaling pathways (Figure 6). Additional bioinformatic analysis evaluated the potential interaction of miRNAs and genes associated with aMCI and AD, which could affect the susceptibility to disease by altering gene expression or regulatory pathways. In this regard, several associated genes were predicted as targets of the miRNAs by TargetScanHuman tools (Supplementary Table 1). In addition, miRPathDB allowed predicting the potential role of these miRNAs into neurodegenerative and neuroinflammatory pathways underlying aMCI and AD (Supplementary Table 1).

## Discussion

In the present study we show that aMCI and sporadic AD are multifactorial conditions resulting from a complex crosstalk among multiple molecular and biological processes, whose perturbation, together with the disruption of compensatory mechanisms ensuring brain homeostasis, may lead to chronic, progressive neurodegeneration (De Strooper and Karran, 2016; Tao et al., 2020). Indeed, the present work highlights interesting insights into the risk factors associated with these conditions (Figure 1A). In particular, the identification of shared variants (rs10466829, *CLECL1*; rs1800795, *IL6*; rs3734050, *FAT2*; rs62182086, *PNKD*; rs11218343, *SORL1*; rs729022, *SYT11*; rs3745453, *ZSWIM4*) supports the existence of “bridge genes” linking both conditions. Indeed, the risk variants displayed slightly higher ORs in AD patients compared to aMCI subjects (Figure 1B). This result suggests that these variants could be predictive of a higher risk of progressing toward AD and support the existence of a risk trajectory linking aMCI to AD, although additional variants are likely to contribute. Overall, the identification of shared variants suggests their potential use for screening patients with aMCI who are at higher risk of developing AD and may need personalized clinical or follow-up treatments.

The use of machine-learning approaches allowed testing the ability of the associated variants in discriminating cases from controls by the use of reliable regression models (Figure 2 and Table 4). These results support the need of further exploring the associated variants in order to identify the genetic determinants that shape the susceptibility for aMCI and sporadic AD and, thus, may be employed for stratifying patients at higher risk of disease, who may benefit of early diagnosis and treatments or different follow-up programs. On this subject, the identification of sex as a predictive variable of differential susceptibility to disease highlighted the importance of developing specific approaches for early treating and monitoring aMCI and AD conditions, taking into account the different prevalence of disease and the environmental factors among male and female patients (Guaita et al., 2015; Nebel et al., 2018).

Among the associated variants, it is interesting to mention the rs3745198 (*PLD3*), rs62182086 (*PNKD*), rs3734050 (*FAT2*, *FAT Atypical Cadherin 2*), which are reported as significant expression Quantitative Loci (eQTLs) in basal ganglia and cortex on Gtex portal^1^. Both regions of the brain are known to be affected by neurodegenerative processes (Ferese et al., 2015; Yacoubian, 2017; Vitanova et al., 2019) and the above-mentioned genes have been associated with neurological disorders mediated by degenerative mechanisms, including LOAD (*PLD3*), spinocerebellar ataxia (*FAT2*, *PLD3*), paroxysmal non-kinesigenic dyskinesia (*PNKD*) (Nibbeling et al., 2017; Tan et al., 2019; Garone et al., 2020). Indeed, these variants could be further explored as potential biomarkers associated with a higher predisposition to develop neurodegenerative disorders, in combination with other contributing factors.

Moreover, a closer look at the genes harboring the variants associated with aMCI and sporadic AD in this study revealed interesting insights concerning the complex molecular and biological processes involved in the pathophysiology of aMCI and AD. Most of the genes associated with aMCI were related to many regulatory mechanisms involved in neuroinflammation, maintenance of cellular homeostasis and response to stress (Figure 3). Such mechanisms are generally mediated by brain cells (i.e., microglia, astrocytes, oligodendrocytes, endothelial), although peripherally-derived immune cells (i.e., monocytes, macrophages and dendritic cells) have also been proposed as participants to inflammatory neuroimmune processes that, ultimately, contribute to neurodegeneration (Bossù et al., 2015; De Strooper and Karran, 2016; Dourlen et al., 2019; Bernaus et al., 2020). The upstream analysis pointed out the attention toward the interaction among aMCI-associated genes and miRNAs and miR-155 and PPARG signaling pathways (Figure 5). Interestingly, both of them are known molecular players taking part in aging processes, neuroinflammatory contexts and cognitive impairment and are under active investigation for therapeutic purposes (Sierksma et al., 2018; D’Angelo et al., 2019; Hemonnot et al., 2019; Juźwik et al., 2019; Senatorov et al., 2019; Bernaus et al., 2020). In addition, the present study highlighted an interaction between miR-146 and miR-155 signaling pathway, which is consistent with literature studies showing their cooperation in the modulation of the microglial inflammatory profile and supporting them as druggable targets for restoring microglial activity in multiple neurodegenerative disorders (Sierksma et al., 2018; Kou et al., 2020; Varma-Doyle et al., 2021). In this scenario, it is important to include even miR499a and miR-196a2, which have been associated with aMCI in this study and with other complex disorders (Kiselev et al., 2015; Strafella et al., 2021a,b).

In addition, some of the genes and miRNAs carrying variants associated with aMCI were enriched in iron homeostasis signaling (*IL6*, *STAT3*, *MAPK1*, *SORL1* and *CYP24A1*), vascular function and proliferation of endothelial cells (*IL6*, *STAT3*, *MAPK1*, *SEMA5A*, *ZMIZ1*, miR-499a and miR146a) (Figure 3). These results suggested the potential role of these genes as contributors to neuroinflammation and neurodegeneration-related events mediated by iron dyshomeostasis or vascular dysfunction, which have been associated with cognitive function and AD pathology (Iturria-Medina et al., 2016; Hemonnot et al., 2019; Ndayisaba et al., 2019; Giannoni et al., 2020). Indeed, both of them represent two of the most promising druggable pathways for developing multi-target therapeutic interventions able to modify or monitor the disease progression (Iturria-Medina et al., 2016; Ndayisaba et al., 2019; Giannoni et al., 2020). In this perspective, the present study supports their therapeutic potential and encourage functional studies aimed at exploring the possible contribution of the above-identified genes for the development of multi-target treatment approaches for MCI.

Concerning AD-associated genes, they were shown to be mostly enriched in neurological disease conditions and functions (Figure 4). The upstream analysis revealed interactions among a set of associated genes (*APOE*, *LRRK2*, *CLU*, *SORL1*, *PLD3*, *IL23R*, *PNKD*) and the APP and ACE2 signaling pathways (Figure 6). Both of them have been extensively investigated in the context of AD complex pathology and neurodegeneration and as potential targets for therapeutic purposes (Kaur et al., 2015; De Strooper and Karran, 2016; Kehoe et al., 2016; Kehoe, 2018; Dourlen et al., 2019). In this regard, the identification of downstream-regulated genes extend the knowledge of these two pathways, providing additional targets to be further investigated for the research of effective multi-target therapeutic interventions for AD.

Overall, this study highlighted that the susceptibility to aMCI condition may be the result of the interaction among genes and miRNAs involved in neuroinflammation, synaptic failure, neuron miswiring, alteration of peripheral immune response, vascular function and iron dysregulation. In this scenario, these genes can affect the homeostasis of both neuronal and non-neuronal cells (i.e., microglia, astrocytes, immune cells, endothelial cells), conferring a higher susceptibility to aging processes, neuroinflammatory and neurodegenerative events. Concerning AD susceptibility, the associated genes and their related interactions confirm the view of the disease as the result of a complex and heterogeneous matrix composed of multiple genetic features, biological pathways, cellular and molecular players which finally disrupt brain function and homeostasis. In-between, the identification of risk variants shared with both aMCI and AD, should be further explored in order to develop personalized approaches in relation to the individual risk profile and disease stage of patients. In this perspective, the above-presented data should be replicated on large-scale studies in order to test their possible use as functional biomarkers to determine the susceptibility to aMCI, the risk of progression toward AD or for developing more effective treatments based on a multi-target approach.

## Data Availability Statement

The original contributions presented in the study are included in the article/Supplementary Material, further inquiries can be directed to the corresponding author/s.

## Ethics Statement

The studies involving human participants were reviewed and approved by Ethical Committee of IRCCS Santa Lucia Foundation of Rome. The patients/participants provided their written informed consent to participate in this study.

## Author Contributions

CS, VC, RC, and EG: conceptualization. VC, GC, DM, PR, and ET: methodology. CS, AT, and CF: software. AT and CF: statistical analysis. NB, AB, and GS: clinical assessment and recruitment of patients. PB, GS, CC, and EG: resources. CS, VC, ET, NB, AB, PB, and GS: data curation. CS: writing-original draft preparation. CS, VC, NB, PB, CC, GS, RC, and EG: writing-review and editing. RC and EG: supervision. CS, EG, and RC: project administration. All authors have read and agreed to the published version of the manuscript.

## Conflict of Interest

The authors declare that the research was conducted in the absence of any commercial or financial relationships that could be construed as a potential conflict of interest.

## Publisher’s Note

All claims expressed in this article are solely those of the authors and do not necessarily represent those of their affiliated organizations, or those of the publisher, the editors and the reviewers. Any product that may be evaluated in this article, or claim that may be made by its manufacturer, is not guaranteed or endorsed by the publisher.

## Footnotes

1
https://gtexportal.org/home/
