# Supplementary material for: Identification of Genetic Networks Reveals Complex Associations and Risk Trajectory Linking Mild Cognitive Impairment to Alzheimer’s Disease
Source: Front Aging Neurosci. 2022 Feb 17;14:821789. doi: 10.3389/fnagi.2022.821789 (PMC8892382; doi:10.3389/fnagi.2022.821789)
Supplement: Supplementary file 1 [file Table_1.DOCX]

Supplementary Material

# Supplementary Table 1. Prediction analysis of the miRNA variants associated with aMCI. aMCI: amnestic Mild Cognitive Impairment.

| **miRNA** | **MCI-associated genes targeted by miRNAs** | **Biological pathways** |
| --- | --- | --- |
| **miR-146-3p** | *IL6, STAT3, SYT11, MAPK1, SEMA5A, IL7R, TNFSF14, ZMIZ1* | Neuroinflammation, neurogenesis, neuron differentiation, angiogenesis, immune response, differentiation and projection of neurons, signal transduction |
| **miR-196a2-3p** | *MAPK1, SYT11, SEMA5A* | Neuroinflammation, neurodegeneration, oxidative stress, Endoplasmic Reticulum Stress, synaptogenesis, endocytosis, Unfolded Protein Response |
| **miR-499a-3p** | *MAPK1, INPP5D, IL7R, SEMA5A, TNFSF14* | Neuroinflammation, neurodegeneration, oxidative stress, Endoplasmic Reticulum Stress, synaptogenesis, endocytosis, Unfolded Protein Response |
